# The Role of IL-6 in Skin Fibrosis and Cutaneous Wound Healing

**DOI:** 10.3390/biomedicines8050101

**Published:** 2020-04-30

**Authors:** Blair Z. Johnson, Andrew W. Stevenson, Cecilia M. Prêle, Mark W. Fear, Fiona M. Wood

**Affiliations:** 1 School of Biomedical Sciences, University of Western Australia, Crawley, WA 6009, Australia; Andrew.Stevenson@uwa.edu.au (A.W.S.); cecilia.prele@uwa.edu.au (C.M.P.); mark.fear@uwa.edu.au (M.W.F.); Fiona.Wood@health.wa.gov.au (F.M.W.); 2 Institute for Respiratory Health, University of Western Australia, Crawley, WA 6009, Australia; 3 WA Department of Health, 189 Royal St, East Perth, WA 6004, Australia

**Keywords:** IL-6, fibrosis, skin, wounds

## Abstract

The timely resolution of wound healing is critical for restoring the skin as a protective barrier. The switch from a proinflammatory to a reparative microenvironment must be tightly regulated. Interleukin (IL)-6 is a key modulator of the inflammatory and reparative process: it is involved in the differentiation, activation, and proliferation of leukocytes, endothelial cells, keratinocytes, and fibroblasts. This review examines the role of IL-6 in the healing of cutaneous wounds, and how dysregulation of IL-6 signaling can lead to either fibrosis or a failure to heal. The role of an IL-6/TGF-β feedback loop is discussed in the context of fibrogenesis, while IL-6 expression and responses in advanced age, diabetes, and obesity is outlined regarding the development of chronic wounds. Current research on therapies that modulate IL-6 is explored. Here, we consider IL-6′s diverse impact on cutaneous wound healing.

## 1. Introduction

Interleukin (IL)-6 plays a central role in acute inflammation and is necessary for the timely resolution of wound healing [1,2]. Released early in response to injury, IL-6 induces the release of proinflammatory cytokines from tissue resident macrophages, keratinocytes, endothelial cells, and stromal cells. IL-6 has also been found to induce chemotaxis of leukocytes into a wound [3,4]. As inflammation progresses, IL-6 signaling is responsible for the switch to a reparative environment. The regulation of wound healing is critical: inappropriate proinflammatory signaling can result in wounds that take much longer to heal and are at risk of infection [5,6,7]. Non-healing wounds can cause distress and require careful management [8,9]. Alternatively, if the switch to proliferative signaling is not carefully controlled, then repair can instead result in fibrosis, which is characterized by the excessive accumulation of extracellular matrix proteins, such as collagen, at the site of injury/damage. Scar formation is the normal end point of mammalian tissue repair, however, excessive scarring can impair normal tissue function [10]. Fibrotic skin tissue covers a spectrum of severity, from flat and pale and relatively static atrophic scars to severe, highly pigmented and rapidly growing pathological hypertrophic and keloid scars [11]. Even the minor normotrophic scar type is dysfunctional—it has decreased sensation, can cause discomfort through itchiness and pain, has altered pigmentation [12], and the tightening of the skin can impair movement and have a detrimental impact on quality of life [13,14]. Scarring also has a psychological toll on patients, due to dissatisfaction with the scar appearance and associated stigma [15]. In this review, we will explore how IL-6 mediates the process of dermal wound repair, and the mechanisms by which it can lead to skin pathology.

## 2. Wound Healing

**Figure 1:**
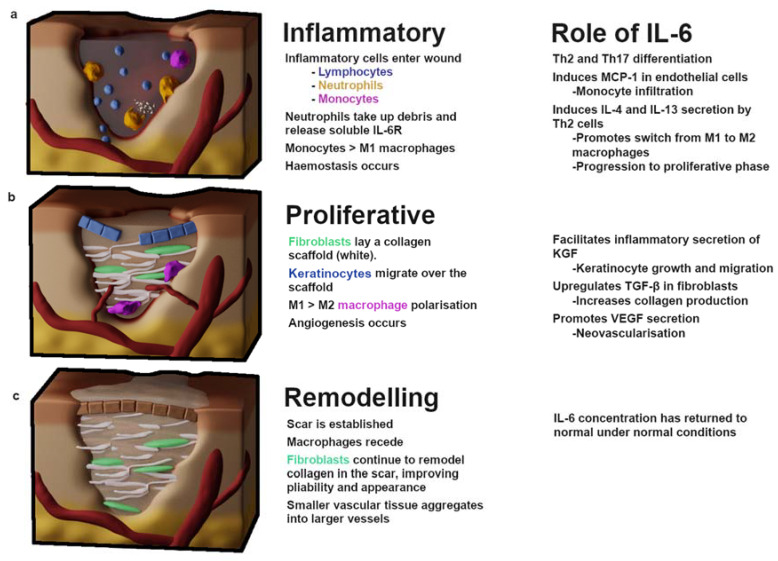
The stages of wound healing. (**a**) The inflammatory phase begins with the influx of platelets and clotting factors which induce hemostasis to prevent blood loss. Inflammatory leukocytes enter the wound—neutrophils clear debris, while lymphocytes, monocytes and tissue-resident macrophages begin differentiating and releasing proinflammatory cytokines IL-1α, IL-1β, IL-6, IL-17, TNF-α, IFN-γ. This phase features predominately M1-type macrophages. (**b**) Proliferation begins with an influx of fibroblasts and TGF-β, resulting in the deposition of a collagen-I scaffold. M1 macrophages polarize towards an M2 phenotype which drives profibrotic signaling necessary for scaring. Keratinocytes migrate over the collagen scaffold and angiogenesis begins. (**c**) Remodeling occurs after a rigid collagen-rich scar has been established; over the course of months to years fibroblasts digest collagen-III and replace it with collagen-I while reorganizing the collagen fibers, which improves scar pliability. IL—interleukin; KGF—keratinocyte growth factor; MCP-1—monocyte chemoattractant protein 1; Th—T-helper; TGF-β—tissue growth factor β; VEGF—vascular endothelial growth factor.

Wound repair has been stratified into many different levels, but there are three primary stages: an inflammatory response, whereby hemostasis is achieved and leukocyte infiltrates clear the wound of debris; a proliferative phase, during which vascularization, extracellular matrix (ECM) deposition and re-epithelialization occur; and remodeling, which may persist for months to years as the ECM is processed and the tissue gains strength and flexibility [16] (Figure 1). In normal wound repair, the expression of IL-6 is significantly decreased during the remodeling phase; this is thought to be due to apoptosis of infiltrating inflammatory leukocytes and a subsequent reduction in cytokine signaling [17]. The role of IL-6 in inflammation is discussed below.

### 2.1. Inflammation

**Figure 2:**
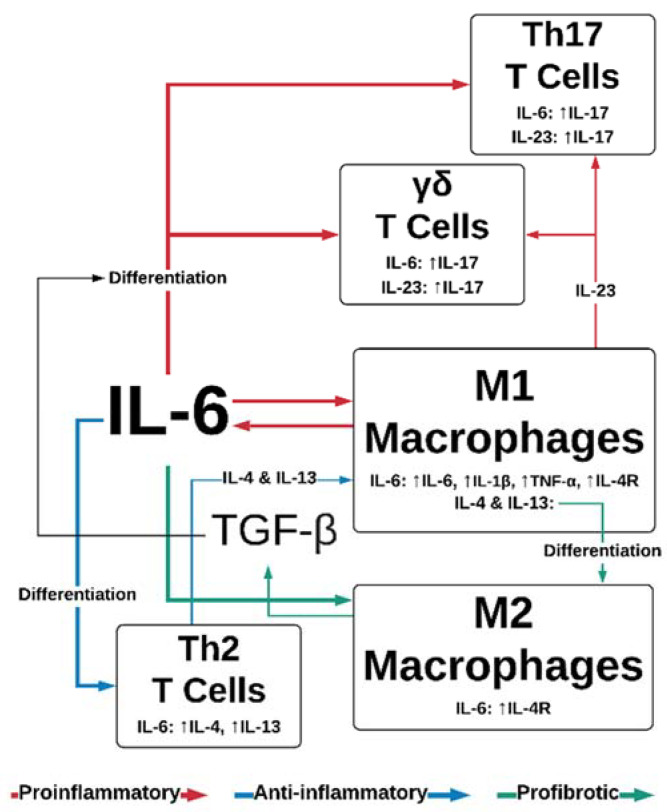
IL-6 and TGF-β effects on leukocyte function. IL-6 promotes the proinflammatory functions of Th17, γδ T-cells, and M1 macrophages, while simultaneously promoting anti-inflammatory Th2 differentiation and cytokine secretion. IL-6 also stimulates profibrotic responses in M2 macrophages, in conjunction with M1/M2 polarization sustained by Th2 cytokines IL-4 and IL-23. TGF-β contributes to IL-6-dependent differentiation of Th17 and γδ T-cells. IL—interleukin; TGF-β—tissue growth factor β; Th—T-helper; TNF-α—tumor necrosis factor α; VEGF—vascular endothelial growth factor.

Tissue injury results in cell necrosis and loss of cell membrane integrity, which causes the release of damage-associated molecular patterns (DAMPs)—molecules, including ATP, DNA fragments and IL-33, IL-1α—that are intracellular under normal physiological conditions, but stimulate immunomodulatory or inflammatory effects when released after cell lysis [18]. DAMPs can stimulate pattern recognition receptors on immune cells, such as formyl peptide receptor-1 and Toll-like receptors (TLRs) on tissue-resident macrophages and infiltrating neutrophils [19,20,21], initiating the proinflammatory NF-κB and mitogen-activated protein kinase (MAPK) pathways. Macrophages secrete proinflammatory cytokines including IL-6, a major regulator of the acute inflammatory response. IL-6 has an array of downstream targets: it promotes Th2 and Th17 differentiation in CD4+ T-cells [22] (Figure 2), while inhibiting the TGF-β-dependent differentiation of T-regulatory cells [23]. Endocrine signaling to the bone marrow results in thrombopoiesis [24], vital for hemostasis. An influx of neutrophils into the wound (recruited via IL-8) initiates the clearing of debris and also introduces soluble IL-6-receptor α (sIL-6Rα) into the wound, as it is shed from the surface of neutrophils [25]. This is an important step in the movement from the inflammatory phase of wound healing to the proliferative phase, as IL-6/IL-6Rα complexes initiate signaling through the ubiquitous gp130 transmembrane receptor, thereby increasing the endothelial expression of IL-6 and monocyte chemoattractant protein 1 (MCP-1) [26]. MCP-1 recruits circulating monocytes, while inflammatory cytokines (tissue necrosis factor alpha; TNFα, and interferon gamma; IFN-γ) and the interaction of DAMPs with TLRs regulate their differentiation into M1 macrophages [27,28]. There is some evidence that the phagocytosis of apoptotic neutrophils towards the end of the inflammatory phase contributes to the macrophage polarization; a switch from the IL-6-producing, proinflammatory M1 phenotype to the TGF-β producing, reparative M2 phenotype [29,30]. This transition is likely bolstered by IL-4 and IL-13, produced by the IL-6 stimulation of Th2 cells [31] (Figure 2).

### 2.2. Proliferation

**Figure 3:**
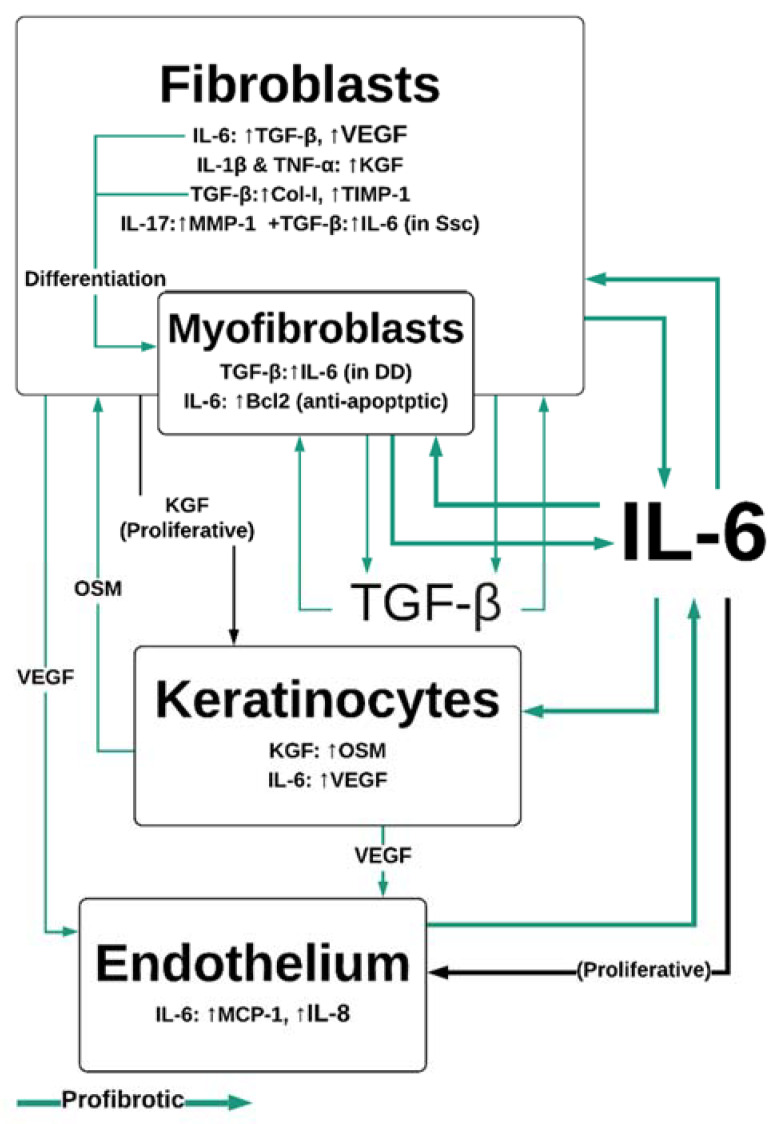
IL-6 and TGF-β-mediated interactions between dermal cells. IL-6 is central to profibrotic interactions between fibroblasts, myofibroblasts, keratinocytes and endothelial cells. IL-6 drives proliferation in endothelial cells in conjunction with VEGF, leading to neovascularization, which sustains pathological fibroproliferative scarring. IL-1β, TNF-α and IL-6 are produced by M1 macrophages in the wound site, while IL-17 is produced by Th17 and γδT-cells. Col-I—collagen 1; DD—Dupuytren’s disease IL—interleukin; KGF—keratinocyte growth factor; MCP-1—monocyte chemoattractant protein; MMP—matrix metalloproteinases; OSM—oncostatin M; SSc—systemic sclerosis; TGF-β—tissue growth factor β; TNF-α—tumor necrosis factor alpha; VEGF—vascular endothelial growth factor.

IL-6 trans-signaling, whereby sIL-6Rα/IL-6 associates with gp130 and initiates JAK/STAT and MAPK signaling pathways [32], stimulates the migration of fibroblasts to sites of injury. Fibroblasts are responsible for producing the collagen and fibronectin scaffold required for re-epithelialization of the wound [5] (Figure 1b). The orientation and composition of this scaffold impacts the outcomes of wound healing. The deposition and remodeling of this matrix is an ongoing process and has been shown to be influenced by growth factors, such as those in platelet-rich plasma, which includes IL-6 and TGF-β (among others) [33,34]. An engineered-tissue model of skin fibrosis demonstrated a dichotomy between the gene expression of IL-6 and of collagen production by superficial and deep dermal fibroblasts in co-culture with keratinocytes [35]. Deep dermal fibroblasts produce significantly more IL-6 than superficial fibroblasts, which may in part contribute to the difference in healing between superficial and deeper wounds [10]. IL-6 signaling helps to initiate profibrotic fibroblast/keratinocyte interactions: IL-6 induction of the proinflammatory cytokine production in macrophage/monocytes occurs via MAPK and NFκB signaling pathways. Monocytes and macrophages secrete IL-1β and TNF-α [36,37] in response to IL-6 exposure, which induces keratinocyte growth factor (KGF) production by fibroblasts [38] (Figure 3). KGF is a potent activator of keratinocytes that enhances their proliferation and migration [39]. Subsequently, keratinocytes produce oncostatin M (another member of the IL-6 cytokine family), which acts in a paracrine manner to stimulate profibrotic STAT3 signaling in dermal fibroblasts [40,41].

IL-6 regulates M2 macrophage polarization, likely by upregulation of the IL-4 receptor [31,42,43,44]; M2 cells are important for late-stage wound repair, expressing TGF-β and IL-10 [45], which are anti-inflammatory. TGF-β is an important activator of the collagen-I pathway in fibroblasts, facilitating ECM deposition, and as an inhibitor of ECM degradation, by promoting the tissue inhibitor of metalloproteinases 1 (TIMP-1) [46]. Wound closure relies on the differentiation of fibroblasts to alpha-smooth muscle actin (α-SMA)-expressing myofibroblasts, which contract to bring the edges of the wound together [47]. IL-6 regulates the differentiation of fibroblasts to myofibroblasts through the paracrine expression of TGF-β at the wound site [45,46], and directly in fibroblasts through the JAK/ERK pathway [48].

Endothelial cells are a direct and indirect target of IL-6/IL-6R/gp130 pathway activation [49,50], which has implications for wound healing. When stimulated with IL-6, endothelial cells are more proliferative and produce MCP-1, IL-8, and IL-6 in a JAK/STAT dependent manner [51], contributing to the inflammatory microenvironment by recruiting leukocytes. Fibroblasts, keratinocytes, and macrophages express vascular endothelial growth factor (VEGF) in response to IL-6 [52,53]. VEGF is one of the primary stimulators of neovascularization [54]. Vascularization is an important component of wound healing, and impaired vascularization delays wound closure [55]. However, the dysregulation of vascularization, potentially through the expression VEGF, may be a component of fibrotic disease pathogenesis, as many fibrotic diseases, much like proliferative neoplasms, feature increased vascularization [56,57].

## 3. Fibrotic Diseases of the Skin

### 3.1. Hypertrophic Scarring

Hypertrophic scars are the result of prolonged inflammation that persists during early wound healing and after wound closure [58,59]. They present as firm, raised scar tissue that may be darker than the surrounding skin due to increased vascularization [56]. Patients describe the scars as being frequently itchy and uncomfortable [11], which is likely due to ongoing inflammation [58]. The risk of hypertrophic scarring is relative to the time a wound takes to heal (up to the remodeling phase); after 21 days that risk rises to 70% [59]. There is evidence that changes to the tension of the dermis during wound healing prolongs inflammation, by promoting vascular permeability [58,60], resulting in increased IL-6 and other proinflammatory mediators within the tissue. The absence of growth factors released by platelets during the first 10 days of wound healing may impede the reorganization of collagen in the wound and contribute to hypertrophy [33]. Aside from the aesthetic issues and discomfort caused by hypertrophic scars, there is a risk of physical impediment, as tightening of the skin prevents normal movement due to scarring around joints. Myofibroblasts have been shown to persist in hypertrophic scars and cause contractures [61,62]. As mentioned above, IL-6 has a role in myofibroblast differentiation, and it may also play a role in myofibroblast persistence. Myofibroblasts from hypertrophic scars are resistant to apoptosis through Bcl2 anti-apoptotic signaling [61]; IL-6 (and the related cytokine IL-11) has been shown to induce Bcl2 expression in fibroblasts from idiopathic primary fibrosis patients [63]. It is through these mechanisms that hypertrophic scars develop and persist following cutaneous tissue damage.

### 3.2. Keloids

Keloids are regions of scar tissue that result from excessive fibrosis following wound closure [64,65,66,67] and can be distinguished from hypertrophic scars, as they grow beyond the boundaries of the original wound in a tumor-like fashion [65]. The exact etiology of the disease is unknown. It is likely that a genetic predisposition contributes to the development of keloids, as they are most often seen in darker skinned patients and there is a degree of heritable risk [65,67]. Epigenetic disparity between keloid fibroblasts and normal fibroblasts from the same donors provides some insight into the mechanism driving fibrosis: there is marked hypomethylation in keloid fibroblasts [67], a trait shared with cancer cells. Interestingly, the promotor region of the JAK1 gene is hypomethylated, suggesting that it would show increased expression. Ghazizadeh et al. confirm this, showing increased expression and phosphorylation of not only JAK1, but also STAT3, RAF1, and ELK1, implicating IL-6 signaling in keloid pathogenesis [66]. Furthermore, keloid fibroblasts demonstrate the increased expression of IL-6, IL-6R, and gp130. The expression of IL-6 may be inherent to a feedback loop, wherein the increased expression of TGF-β [64] promotes IL-6 production via PI3K and p38-MAPK [46,68], and IL-6 promotes TGF-β expression by keloid-resident macrophages [69]. As with other fibrotic conditions, these macrophages demonstrate M2 polarization. TGF-β upregulates the expression of VEGF by keloid fibroblasts [64], a key component promoting the vascularization of keloid scars, that allows them to grow excessively.

### 3.3. Scleroderma and Systemic Sclerosis

Scleroderma is an autoimmune disease; however, the exact etiology is poorly understood. There are two broad classifications of the disease: scleroderma refers to a localized thickening and hardening of the skin, whereas the diffuse disease, that may affect visceral connective tissue, is termed systemic sclerosis. Patients may have progressive loss of function as the skin around joints becomes hard and immobile, or internal organs fail due to excessive fibrosis [70,71]. There is some suggestion that the pathogenesis of the disease is rooted in a dysregulated Th17 response and research supporting this demonstrates increased circulating and/or infiltrating Th17 cells in systemic sclerosis. This may be accompanied by elevated IL-17A [72,73,74]; there is, however, some disagreement [75]. In a bleomycin model of cutaneous fibrosis, mice that were highly susceptible to fibrosis due to an IL-1 antagonist deficiency were protected from fibrosis by IL-17 knockdown [76]. This evidence supports IL-17 as a disruptive cytokine potentiating pathological fibrosis. The action of IL-17A in systemic sclerosis is complex: studies have demonstrated that IL-17A (in conjunction with TNF) can be anti-fibrotic through the upregulation of matrix metalloproteinases 1 (MMP-1) and the inhibition of collagen synthesis in human dermal fibroblasts [46,77], although this may not be as effective in fibroblasts isolated from disease sites. Conversely, IL-17A acts synergistically with TGF-β to increase the expression of IL-6 in fibroblasts cultured from systemic sclerosis patients [46]. IL-6 expression is constitutively higher in systemic sclerosis fibroblasts compared to fibroblasts from healthy donors [46]. This suggests that there is a presiding proinflammatory state driving progressive collagen deposition, leading to the stiffening of the ECM. Furthermore, environmental stimulation may contribute to IL-6-dependent fibrosis. For example, Uehara et al. provide data that links norepinephrine and IL-6 production, showing a significantly increased response in scleroderma fibroblasts via adrenoreceptor (AR)β signaling [78]. The ensuing production of collagen could be impeded by the addition of propranolol, an ARβ antagonist, or SB203580, a p38 inhibitor. The authors suggest that increased norepinephrine circulation due to cold temperatures or emotional stress may contribute to fibrosis in systemic sclerosis patients [78].

### 3.4. Dupuytren’s Disease

Dupuytren’s contracture is a progressive fibrotic disease that manifests as nodules of tissue under the surface of the palm. These lesions are contractile and over time cause a tightening of the skin that results in persistently bent fingers. Dupuytren’s contracture is mainly prevalent in individuals over 60 years old; representing around 50% of all diagnoses [79]. The exact etiology of the disease is unknown, however there is a heritable risk [80].

Myofibroblasts from Dupuytren’s nodules are highly proliferative, and express high levels of TGF-β, IL-1β, α-SMA and IL-6 [81]. Normal (non-disease) cells collected from the palmar fascia adjacent to a Dupuytren’s nodule have been shown to express IL-6 upon stimulation with TGF-β, and primary fibroblasts grown from these tissues express IL-6R [82]. This allows an IL-6 autocrine feedback loop to perpetuate proliferative signaling in Dupuytren’s fibroblasts. IL-6 does not appear to control the contractibility of myofibroblasts [81]; this is regulated by TNF. Macrophages have a role to play here too, as TNF is primarily secreted by macrophages. The number of macrophages in the disease tissue is correlated with the presence of myofibroblasts [83], which are indicative of active disease. As with other fibrotic conditions, the growths show increased vascularization [57]. The pro-survival, pro-proliferative signaling of IL-6 and its downstream effector cytokines likely contribute to the microenvironment conducive to angiogenesis.

### 3.5. Treatments Modulating IL-6 (Cutaneous Fibrosis)

The control of IL-6, alongside other inflammatory cytokines, has proven to be an appealing strategy for treating fibrotic conditions (Table 1). Ameliorating low level chronic inflammation perpetuated by, and contributing to, IL-6 expression in fibrotic tissue, may slow or stop the progression of the disease, or even reverse established fibrosis.

#### 3.5.1. Corticosteroids

Corticosteroids have been used to treat hypertrophic scars and keloids for over half a century, with mixed success [99]. Oral administration of corticosteroids has been phased out in favor of local injection and topical application. Triamcinolone acetonide is commonly used for the treatment of early stage Dupuytren’s, hypertrophic and keloid scars [84,85,86,87,88,100], although a range of corticosteroids have been implemented in various treatments for fibrotic conditions [86,101]. Triamcinolone acetonide has been specifically shown to decrease proliferation and IL-6 expression in lung fibroblasts [102], and it inhibits IL-6 and VEGF-dependent angiogenesis in corneal endothelial cells via decreased expression of STAT [103]. It is likely that triamcinolone acetonide reduces cutaneous fibrosis via these mechanisms—the reduction of IL-6 expression would moderate TGF-β-dependent collagen production and limit the neovascularization required to sustain fibroproliferative growth.

Corticosteroids, in combination with other pharmacological or physical therapies, have been shown in a number of studies to have a favorable result in scars. Kant et al. demonstrate corticosteroids combined with verapamil result in a 42% decrease in scar score (patient and observer scar assessment scale [104]) for keloids, and a 33% decrease for hypertrophic scars after 12 months [84]. Park et al. show the use of a topical corticosteroid combined with laser ablation produced a 39% reduction in keloid scar score (Vancouver scar scale [105]), compared to 47% for intralesional injections (however, patients reported significantly less pain with the topical treatment) [86]. On et al. demonstrated that four treatments with triamcinolone injections and a copper bromide laser was sufficient to produce a 50% reduction in Vancouver scar scale score in the majority of patients, with a hypertrophic scar following thyroidectomy [85]. In Dupuytren’s disease, corticosteroids improve the consistency of nodules by 60%–80% within an average of six months, however, reactivation has been shown to occur in 50% of patients within three years, necessitating further treatment [87]. Recurrence of the disease following corticosteroid treatment appears to be improved in non-Caucasians according to a study treating patients in Taiwan [88]. Long-term treatment with corticosteroids appears to be well tolerated if administered according to guidelines (injections at least one month apart) [85,86,87,88]. Corticosteroids can be an effective treatment in fibrotic diseases, although this efficacy is still limited.

#### 3.5.2. Verapamil

Verapamil is a calcium-channel antagonist that has been studied for the treatment of keloids. It limits nodule growth by decreasing the expression of IL-6 and VEGF in keloid fibroblasts, and increasing the rate of fibroblast apoptosis [89]. Calcium has a significant role in the control of wound healing [106], however verapamil is typically administered intralesionally after re-epithelialization. Wang et al. provide a summary of clinical trials using verapamil to treat hypertrophic scars and keloids, noting that the effectiveness was moderately poorer than corticosteroid treatment, however there were fewer adverse effects [90]. These findings are disputed in more recent studies [91,92], suggesting further trials are needed to establish the efficacy of verapamil for the treatment of scars.

#### 3.5.3. IL-6 Blockade

The IL-6Rα antagonist tocilizumab has been investigated as a potential treatment for systemic sclerosis. The faSScinate phase II randomized controlled trial compared the effects of subcutaneous tocilizumab injections on skin fibrosis, compared to controls [93]. It was found that tocilizumab treatment significantly improved the modified Rodnan skin score [94] of patients, compared to placebo controls in the 48 week double-blind period of the study, which was further corroborated when placebo patients were subsequently administered the drug up to week 96. Tocilizumab treatment was associated with an increased risk of serious infections, which is consistent with immunosuppression following the IL-6 blockade [93]. The researchers expanded their investigation to study the effects of treatment on dermal fibroblasts isolated from treatment group patients, placebo-control patients, and healthy controls. They report that tocilizumab treatment had effectively reversed the TGF-β-induced molecular and genetic phenotype observed in systemic sclerosis fibroblasts [95]. There is potential for tocilizumab to be used as a treatment for other fibrotic conditions, although considerations must be made for its immunosuppressive effects.

#### 3.5.4. Pirfenidone

The treatment of Dupuytren’s is still primarily focused on physical disruption or removal of the nodule, although collagenase and steroid treatments are also popular [107,108,109], but even with these treatments, recurrence rates are still high. The TGF-β/IL-6 feedback loop may be controlled by pirfenidone, which reduces the TGF-β-dependent phosphorylation of AKT and p38 in fibroblasts cultured from Dupuytren’s lesions [96]. The efficacy of pirfenidone treatment for idiopathic lung fibrosis (IPF) has been assessed in mouse models, where the lung concentrations of IL-6 and other inflammatory cytokines were ameliorated [110]. In clinical trials, pirfenidone was found to improve progression-free survival up to 52 weeks in patients with IPF [111,112], with good tolerability, and overall survival had been improved at two, three, and five years [113,114,115]. Pirfenidone has anti-fibrotic effects on Dupuytren’s disease derived fibroblasts [96,97,115,116], and appears to be a suitable candidate for clinical trials in patients with Dupuytren’s disease.

## 4. Age, Obesity, Diabetes and Chronic Wounds

Complications with wound healing and fibrosis are seen more frequently in some populations, including the elderly, the obese, and those with diabetes. IL-6 plays a role in this pathology. Here, we describe the pro-inflammatory role of IL-6 and how it can delay wound closure.

### 4.1. Age

IL-6 and other proinflammatory cytokines are upregulated in monocytes in the elderly [117]. However, macrophage infiltration into wounds is delayed [118], prolonging the early inflammatory phase of healing. Fibroblasts become quiescent with age, with decreased motility and proliferative capacity [119]. This is concurred in the wounds of aged mice, which have demonstrably delayed healing and decreased fibroblast-myofibroblast differentiation [120]. This appears to be predominately driven by the reduced expression of TGF-β and the reduced sensitivity to TGF-β-induced signaling in fibroblasts from aged mice [120]. This begs the question: how does an increase in IL-6 expression relate to a decrease in TGF-β expression, given that IL-6 trans-signaling is prostimulatory for TGF-β production? It is possible that decreases in the concentration of soluble IL-6Rα observed in elderly patients [121] impairs IL-6/IL-6Rα activation of gp130 on macrophages, keratinocytes, fibroblasts and endothelial cells. This may contribute to the persistence of a M1 phenotype and delayed transition to the pro-proliferative M2 phenotype [122]. Elderly people may have chronic wounds due to increased IL-6, but show reduced proliferation and scarring due to decreased expression of pro-proliferative, pro-fibrotic TGF-β [123].

### 4.2. Diabetes and Obesity

While there is no apparent correlation between serum IL-6 levels and age [124], elevated circulating concentrations of IL-6 are commonly observed in obesity and diabetes [5,6], as a symptom of chronic low-grade inflammation. Interestingly, a reactive increase in IL-6 expression during acute inflammation is delayed in a mouse model of diabetes [5]. This sluggish inflammatory response contributes to delayed wound closure. Hyperglycemia (a hallmark of diabetes) has been demonstrated to decrease the basal levels of SOCS3 (an endogenous negative regulator of IL-6-induced STAT signaling), while increasing nuclear phosphorylated-STAT3 in keratinocytes [125]. Keratinocytes are subsequently hyperproliferative [126], however, as they do not have increased migratory capacity [125], this is consistent with delayed wound closure. The expression of IL-6 and IL-6Rα is increased in the wound site, contributing to prolonged inflammation [125]. While cutaneous wounds have a tendency to be chronically non-healing, diabetes patients are at the risk of visceral fibrotic disease, including cardiovascular, liver and renal sclerosis [127]. IL-6 has been implicated in the progression of these morbidities [128,129,130], demonstrating the complex role of IL-6 in regulating pro-inflammatory and pro-fibrotic processes.

Obesity has a similar pathology to diabetes, which is unsurprising, as diabetes is a complication that occurs in the obese [131]. However, obesity does present some unique pathophysiology. Adipose tissue is a secretory organ and there is an increase in both the secretion of IL-6 and expression of IL-6Rα in obese patients compared to lean individuals [132]. Thus, they are both a perpetrator of, and responsive to, proinflammatory signaling. Adipocytes are not the major producers of IL-6 in adipose tissue. Instead, IL-6/IL-6R expression correlates with the macrophage-distinguishing markers cluster of differentiation (CD)11b and CD163, as well as macrophage-type cytokines TNF-α and MCP-1 [132]. This is consistent with the histology of pathological adipose tissue: macrophages are seen to form crown-like structures around dead adipocytes [132,133]. These macrophages are typically of the M1 phenotype [134,135], although Braune et al. suggest that M2 macrophages predominate the tissue and obesity leads to a change in M1/M2 polarization towards M1 cells [42].

Aside from the mechanisms that drive the IL-6-dependent delaying of wound closure discussed above, additional mechanisms have been explored. In a mouse model of diabetic obesity, Lee et al. demonstrated increased frequencies of Th17 and IL-17-producing γδT-cells in full-thickness cutaneous wounds, compared to controls [136]. Furthermore, Th17 and γδT-cell frequency was greater in older obese mice compared to young obese mice. IL-6 has been shown to promote Th17 and γδT-cell differentiation in combination with TGF-β [22,137]. These two cell populations produce IL-17 upon stimulation with IL-23 [138,139], another proinflammatory M1 cytokine [140]. Lee et al. provide evidence showing that the inhibition of IL-17 or IL-23 improves wound closure times in diabetic obese mice without impacting scarring or fibrosis [136]. Inhibition of IL-17 also promotes polarization from proinflammatory to pro-repair macrophages. This links IL-6 stimulation of T-cells with pathological wound healing in obesity and diabetes.

The mechanisms above may provide some explanation for the pathology presented in a case study published by Nicoletti et al. [141] Here, a formally obese patient suffered wound dehiscence following breast reduction surgery. It was demonstrated that fibroblasts cultured from this patient were inexplicably torpid compared to fibroblasts cultured from other patients. It is possible that the constitutive inflammatory environment established by obesity contributed to the delay in wound closure, and furthermore impeded fibroblast proliferation.

### 4.3. Treatments Modulating IL-6 (Wound Healing)

Targeting inflammation with corticosteroids is not a robust treatment method for chronic wounds and may only benefit a portion of patients, where others may show worsening pathology [142]. There is disagreement over the effectiveness of corticosteroids in patients that show improving wounds, compounded by a lack of controlled trials [143]. There is generally a lack of clinical research into therapies targeting IL-6 inhibition in chronic wounds; given that IL-6 is known to be important for wound closure [1], this is understandable. Most conventional treatments for chronic wounds aim to use a physical barrier that provides an environment conducive to healing, and may involve the administration of topical growth factors [9,144]. There may be potential for IL-6 to be targeted in a combination treatment with growth factors, whereby the inflammation is controlled without hindering the proliferation of fibroblasts, keratinocytes and endothelial cells. One such treatment encapsulated LL37, an antimicrobial that increases IL-6 and VEGFα expression, with poly lactic-co-glycolic acid (PLGA), a biodegradable supply of angiogenic lactate [145]. These nanoparticles significantly improved in vitro and in vivo (mice) wound closure times. LL37 has also been used in conjunction with the elastase inhibitor serpin A1, which also demonstrated the improved closure of in vitro wounds through promoting fibroblast and keratinocyte mobility [146]. There is potential for these nanoparticles to prevent pathological wound healing by improving wound closure time if applied close to the time of injury. An extensive study would be required to determine if this is viable for the treatment of chronic wounds.

## 5. Conclusions

The role of IL-6 in the healing of cutaneous wounds cannot be understated, and the timing of the inflammatory response is paramount to a successful resolution and wound closure, where impairment of the IL-6 signaling pathway delays wound healing. The primary expressors of IL-6 in the wound are M1 macrophages, and an increase in the M1:M2 ratio, as seen in the elderly and the obese, can be detrimental to wound healing, due to chronic inflammatory signaling. Conversely, IL-6 has some control over M2 polarization through the promotion of IL-4R on macrophages and IL-4 secretion by Th2 lymphocytes, and these M2 macrophages are prominent secretors of the proliferative cytokines TGF-β and VEGF. The IL-6/TGF-β feedback loop is implicated in the pathogenesis of several profibrotic skin conditions, as it operates in a positive autocrine loop in fibroblasts, bolstered by IL-17A, to increase collagen deposition and the differentiation of fibroblasts to myofibroblasts, which contract to pull the edges of a wound together. Stimulation with IL-6 promotes the survival of myofibroblasts, and excessive contracture is a feature of some cutaneous fibropathies. VEGF expression by endothelial tissue, keratinocytes, fibroblasts, and macrophages in response to IL-6 contributes to fibrotic diseases, which rely on increased vascularization. Targeting the IL-6/TGF-β feedback loop with pirfenidone is effective in treating IPF, and tocilizumab reduces cutaneous fibrosis in systemic sclerosis. These drugs have potential as treatments for fibrotic skin diseases. The pleiotropic function of IL-6 in cutaneous pathology is complex, and its diverse potential as a proinflammatory, profibrotic and antifibrotic cytokine deserves further investigation, with significant potential for further developments in clinical intervention.

## Figures and Tables

**Table 1:** Treatments that modulate IL-6 in cutaneous fibrosis.

Treatment	Effect	Disease	Outcome
Corticosteroids	↓IL-6, ↓VEGF, ↓STAT3	Hypertrophic/keloid scars	Improved scar score [84,85,86]
		Dupuytren’s disease	Softening of nodules, improved motility [87,88]
Verapamil (Calcium antagonist)	↓IL-6, ↓VEGF, ↑Apoptosis (Fibroblasts)	Hypertrophic/keloid scars	Reduced growth, improved scar score [89,90] (disputed efficacy [91,92])
Tocilizumab (IL-6 blockade)	Prevents IL-6-receptor binding	Scleroderma/systemic sclerosis	Improved skin score, reverses TGF-β-dependent phenotype in SSc fibroblasts [93,94,95]
Pirfenidone	↓TGF- β-signaling, ↓IL-6	Dupuytren’s disease	Preclinical—reduces TGF-β-dependent signaling in DD fibroblasts [96,97,98]

SSc—systemic sclerosis; DD—Dupuytren’s disease.
